# Visualizing and teaching crystallographic symm­etry using *Jmol*

**DOI:** 10.1107/S2053229626004559

**Published:** 2026-05-18

**Authors:** Dean H. Johnston, Robert M. Hanson

**Affiliations:** a Department of Chemistry, Otterbein University, Westerville, OH, USA; b St Olaf College,Northfield MN USA; University of Strathclyde, United Kingdom

**Keywords:** Jmol, Space Group Symmetry Visualizer, crystallographic symm­etry, visualization

## Abstract

The *Jmol***Space Group Symmetry Visualizer** website provides a modular set of interactive pages for the visualization of symmetry groups, including both space groups and plane groups, as well as subperiodic groups (frieze, rod, and layer). Groups and group–subgroup relationships are illustrated using a variety of molecular and ionic structures.

## Abstract 2

The *Jmol***Space Group Symmetry Visualizer** (https://spacegroups.symotter.org) is an online resource for the visualization of crystallographic symm­etry built around the versatile *Jmol* application and represents a unique resource for students and educators in crystallography and researchers using crystallographic methods.

## Introduction

The *Jmol***Space Group Symmetry Visualizer** (https://spacegroups.symotter.org) is an online resource for the visualization of crystallographic symm­etry built around the versatile *Jmol* application. This website provides a modular set of inter­active pages for the visualization of sym­metry groups, including both space groups and plane groups, as well as sub­periodic groups (frieze, rod, and layer). Groups and group–subgroup relationships are illustrated using a variety of mol­ecular and ionic structures. All symm­etry elements are color-coded and can be selectively dis­played for easy com­parison to conventional space group diagrams. Wyckoff positions are pre­sent­ed in tabular form, and representations can be generated and inter­actively dragged, maintaining their respective symm­etry relationships. Atoms in structures are listed by Wyckoff position and can be labeled and colored by element, Wyckoff, or site symm­etry. Maximal subgroups for all space groups are listed, and representations of group–subgroup relationships are easily generated. Taken together, these capabilities represent a unique resource for students and educators in crystallography and researchers using crystallographic methods.

## Resources for teaching crystallographic symm­etry

The challenge of teaching crystallographic symm­etry is evi­dent considering the numerous resources that have been created and the variety of approaches described in the literature (Enemark, 1988 ▸; Cooper *et al.*, 1989 ▸; Hardgrove, 1997 ▸; Boo & Mattern, 2008 ▸; Pett, 2010 ▸; Chapuis, 2011 ▸; Gražulis *et al.*, 2015 ▸; Ruiz & Johnstone, 2020 ▸; Dong & Zheng, 2021 ▸; Zheng & Campbell, 2021 ▸; Kotsis, 2025 ▸).

### Two-dimensional symm­etry diagrams and tutorials

Teaching crystallographic symm­etry requires the communication of three-dimensional concepts using primarily two-dimensional representations. A common approach is to use the two-dimensional plane groups to introduce symm­etry concepts and terminology that can later be extended to space groups (Brady, 1978 ▸; Stróż, 2003 ▸; Duda *et al.*, 2020 ▸). Plentiful examples from textiles and the decorative arts (Glasser, 1967 ▸; Hargittai & Lengyel, 1984 ▸; Hargittai & Lengyel, 1985 ▸; Schoeni *et al.*, 2013 ▸) provide an accessible and engaging approach for students at all levels. The advent of personal com­puters allowed the creation of pro­grams to display space group diagrams and mol­ecular representations (Penfold & Temple, 1982 ▸; Temple, 1985 ▸). As com­puters became more capable, software like Margaret Kastner’s pioneering *Crystallographic Courseware* (Kastner, 1999 ▸; Kastner *et al.*, 2000 ▸; Kastner *et al.*, 2013 ▸) provided instructional material covering many topics, including symm­etry, asymmetric units, plane groups, and reading the Inter­national Tables for Crystallography (Aroyo, 2016 ▸). In a similar vein, the excellent *Symmetry and Space Group Tutorial* developed by Bruce Foxman and Jerry Jasinski (Foxman, 2021 ▸) utilizes careful step-wise building up of space group diagrams and occasional humor to create an effective and enjoyable tutorial. The recently developed GESUS (Grupos Espaciales de Simetria de la Universidad de Sevilla) application provides an inter­active environment where students can learn symm­etry operations and conventional representations through a set of tutorial exercises (Miras *et al.*, 2022 ▸).

### Three-dimensional physical models

The use of physical models has a long history in crystallography and structure determination, so it is not surprising that models, ranging from ball-and-stick to papercraft to 3D-printed objects, have been employed for teaching space groups, Bravais lattices, and crystal forms (Hathaway, 1979 ▸; Sein & Sein, 2015 ▸; Graw & Stalke, 2022 ▸; Aristov *et al.*, 2022 ▸). The opportunity for students to build or physically inter­act with models can lead to insights and understanding about how mol­ecules are arranged in unit cells and their symm­etry relationships. Creative use of inexpensive materials makes these models accessible for a wide range of audiences.

### Three-dimensional com­puter representations

With the advent of high-quality color com­puter graphics displays, the power and potential to display three-dimensional crystal structures and space group symm­etry was realized by several groups (Abad-Zapatero & O’Donnell, 1987 ▸; Sakurai *et al.*, 1989 ▸) and eventually became part of commercial software packages (Khosrovani *et al.*, 1999 ▸). As personal com­puters have become more powerful, increasingly com­plex images and videos have been produced (De Graef, 1998 ▸; De Graef, 2009 ▸; Bucio *et al.*, 2024 ▸) to support the teaching of crystallographic symm­etry. Other educators have embedded three-dimensional figures of the 32 crystallographic point groups into PDF files (Arribas *et al.*, 2014 ▸) and created animations and videos for teaching space group symm­etry (Bucio *et al.*, 2024 ▸). Hitzer and Perwass have created the *Space Group Visualizer* (Hitzer & Perwass, 2006 ▸; Hitzer & Perwass, 2021 ▸; Hitzer *et al.*, 2010 ▸; Müller, 2021 ▸), a stand-alone com­puter pro­gram (Windows) for the three-dimensional visualization of space groups and their associated symm­etry operations. More recently, com­puter models in virtual reality environments have been used to introduce space group symm­etry, including the overlay of symm­etry element representations on crystal structures (Graw *et al.*, 2022 ▸; Graw *et al.*, 2023 ▸; Mercado, 2025 ▸).

### Mol­ecular visualization software

Numerous mol­ecular and crystal structure viewing pro­grams developed over the years include the capability to display crystallographic symm­etry information (de la Flor *et al.*, 2024 ▸). Some examples include *MAGE* (Pavkovic, 2005 ▸), *Diamond* (Wu, 2024 ▸), *VESTA* (Momma & Izumi, 2011 ▸), *Mercury* (Macrae *et al.*, 2020 ▸; Battle *et al.*, 2010 ▸), and *Jmol* (Hanson, 2010*a* ▸; Hanson, 2010*b* ▸; Hanson, 2017 ▸). Within this set of pro­grams, *Jmol* occupies a unique position due to its extensive capabilities, flexibility, and scripting language. Originally developed as an open-source mol­ecular viewing pro­gram written in Java, work by Howard starting in 2002 added browser applet capability that allowed integration of the *Jmol* applet into web pages (Howard *et al.*, 2010 ▸). Significant revisions and continued work by Hanson has added extensive crystallographic capabilities to *Jmol*, including reading of crystallographic (CIF) data files, display of unit cells and symm­etry elements, and com­prehensive space group information, all in a well-documented scriptable environment (de la Flor *et al.*, 2024 ▸; Hanson, 2025*a* ▸; Hanson, 2025*b* ▸). *Jmol* is also unique in that the same codebase produces both the stand-alone Java pro­gram (Jmol.jar) and a JavaScript-based web application (sometimes referred to as *JSmol*, but herein referred to simply as *Jmol*) that can be embedded into any web page.

In this article, we will focus on the development and implementation of a browser-based space group symmetry visualizer, demonstrating how *Jmol* can serve as the foundation for a dynamic, inter­active, and effective tool for visualizing and teaching crystallographic symm­etry.

## Design principles

When designing and implementing a website for teaching and sharing mol­ecular and crystallographic symm­etry, our efforts focused on making materials that are accessible, intuitive, and modular. The features of *Jmol* make it possible to create materials that meet all of these goals.

By considering accessibility, we seek to lower the barriers to use by students and implementation by instructors. Accessibility in this context refers to electronic materials that have no requirements beyond a web browser. The visualizations and controls are designed to be effective on a range of devices from laptops to mobile devices and run independent of any other software or applications. Additionally, web pages are designed to be *responsive*, adapting to screen size and orientation. Download sizes for web pages are minimized, taking advantage of the modular structure of *Jmol*, which loads required com­ponents dynamically as needed.

Intuitive design, while more subjective, focuses on creating a resource where most actions can be com­pleted by selecting options from a menu, list, or checkbox. The layout follows that of common websites and apps using conventional elements for buttons and other controls. The inter­face is relatively simple, with the majority of the screen dedicated to the *Jmol*-based inter­active display of space group and mol­ecular representations. All representations are color-coded with legends that clearly indicate all aspects of the diagrams. Google’s Material Design (Google, 2025 ▸) is used throughout to provide an accessible and familiar inter­face.

The modular aspect of these materials makes the site adaptable to a wide range of objectives. This design choice allows the various modules to be tailored for audiences at different levels. There is no pre-determined path or order in which to use the materials, so students and instructors are free to adapt them to their individual needs and environments. The menu-driven selections and inter­active click-and-drag visualizations are intuitive and accessible, thereby appropriate even at an introductory level. For those students and instructors who wish to explore beyond what is exposed by the inter­face, the *Jmol* scripting environment is always available from a pop-up scripting console. Additionally, users can construct representations and share them as a ‘pngj’ file, a standard PNG image format that also contains the full *Jmol* state, allowing the image to be drag-dropped into the *Jmol* Java application or other web pages that utilize *Jmol*.

## Website overview

The site is organized into two modules: (1) the symm­etry group visualizer, and (2) the crystal structure symm­etry visualizer. A large toggle button at the top of the page switches between the two modules.

### Symmetry group visualizer

**Figure 1:**
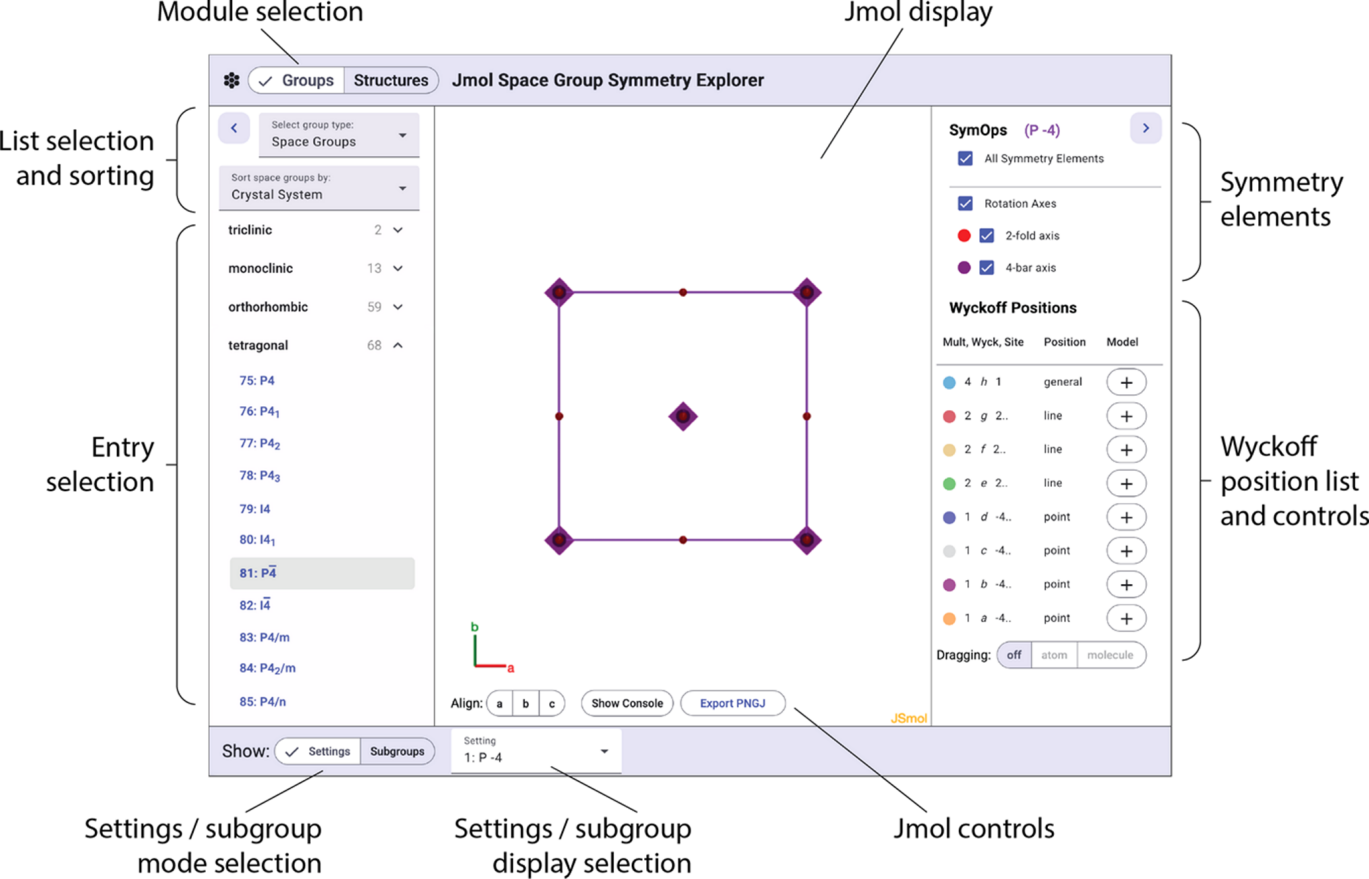
An annotated view of the *Jmol***Space Group Symmetry Visualizer** page displaying the *P*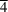 space group and its respective symm­etry elements and Wyckoff positions.

The symm­etry group visualizer is arranged into three panels, as shown in Fig. 1 ▸. On the left are controls for selecting and sorting the group list along with the list itself. Available symm­etry groups include space, plane, frieze, rod, and layer groups. Lists can be sorted by system, class, or number. Selecting a sym­metry group displays a three-dimensional representation of the group in the center panel that at first glance appears to be simply a colorized version of the standard plane-group representations appearing in textbooks and tables of space groups. But clicking and dragging in the center panel rotates the representation. The right panel displays a list of symm­etry elements and a table of Wyckoff positions. The display of symm­etry elements can be toggled on and off using the respective checkboxes. The Wyckoff table includes buttons that allow the user to add and delete pseudo­atoms representing the respective Wyckoff positions in the unit cell. Toggling the **dragging** option at the bottom of the list to **atom** allows these atoms to be dragged in three-dimensional space. All the symm­etry-related atom positions will update dynamically. Atoms in special positions can only be dragged within the constraints defining their Wyckoff position – a plane, axis, or fixed point. Attempts to drag any atom – special or general – to a different Wyckoff position will not be successful, and a graphical warning symbol will be generated.

**Figure 2:**
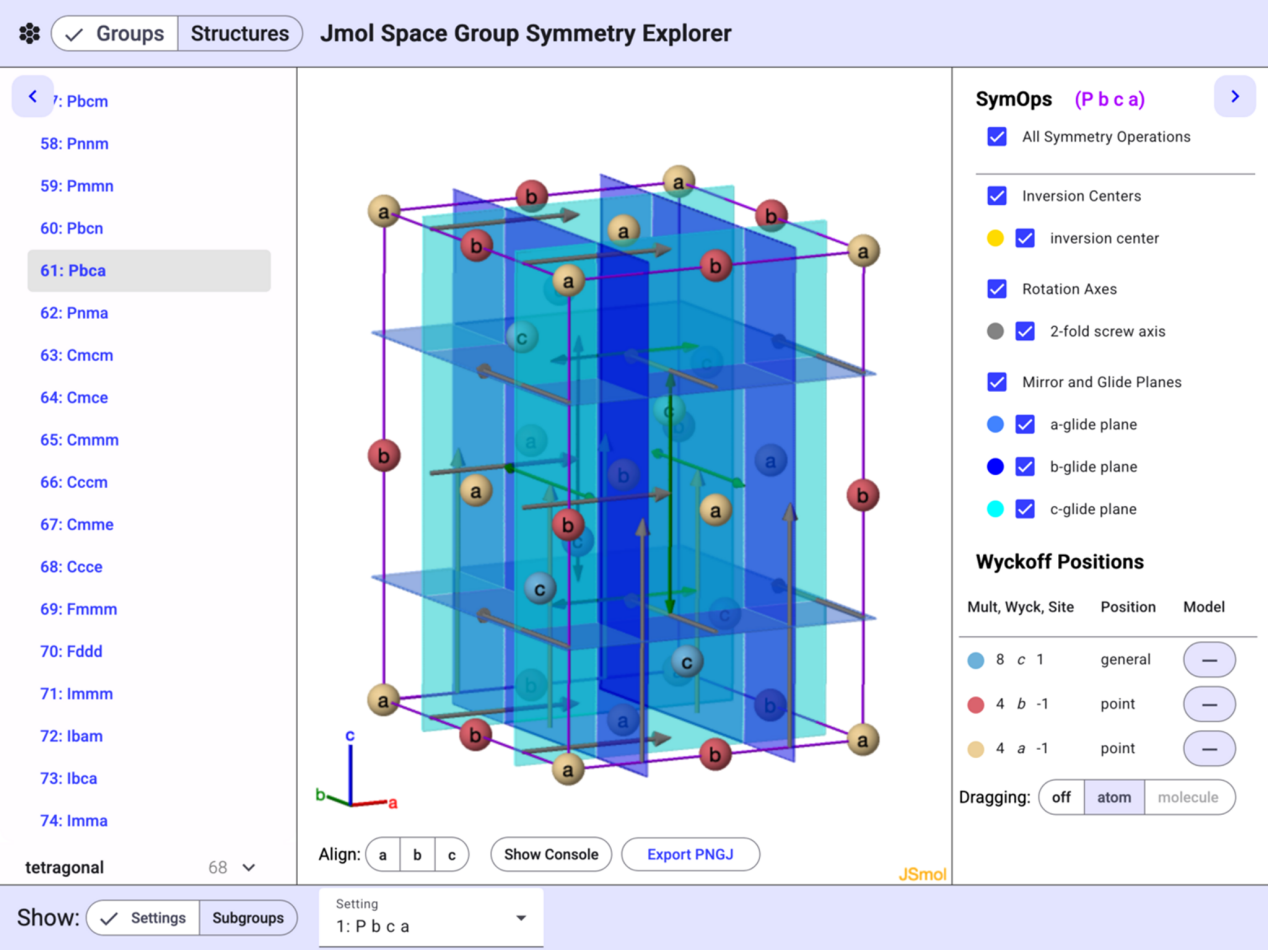
An illustration of the symm­etry elements and Wyckoff positions of the *Pbca* space group.

A more detailed example of the visualizations made possible by *Jmol* is shown in the *Pbca* space group example found in Fig. 2 ▸. The inversion centers, twofold screw axes, and glide planes are all illustrated in the three-dimensional representation with controls and a color-coded key shown on the right side. *Jmol* differs from other pro­grams such as *Mercury* in that it uses unique colors for each of the different types of glide planes and different colors for screw axes of opposite handedness as well as n-bar axes. Representations of all the Wyckoff positions have been added and are shown as colored spheres with labels.

#### Space group settings

**Figure 3:**
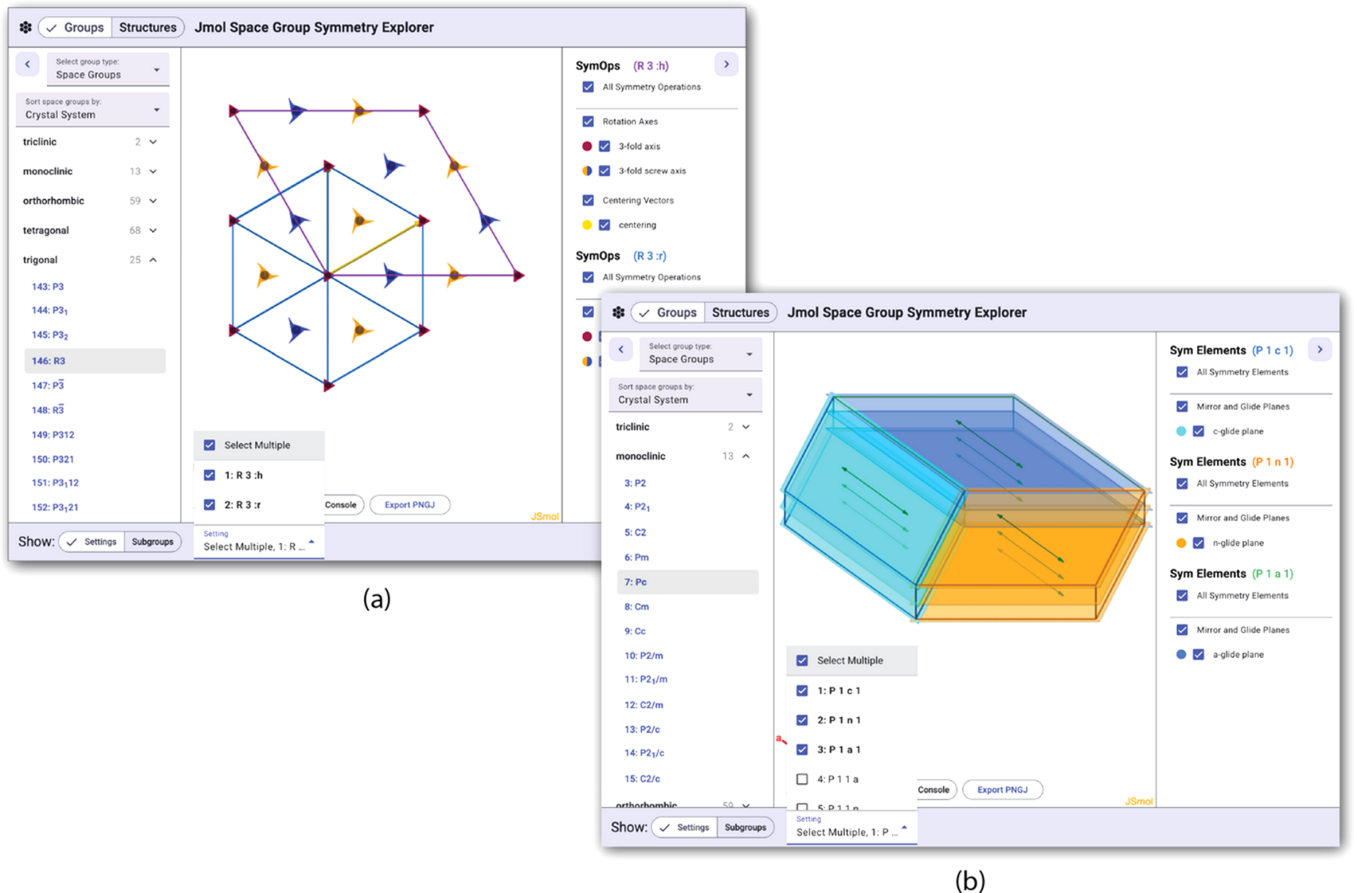
Examples of the display of multiple settings for two different space groups: (*a*) the hexa­gonal and rhombohedral settings of the *R*3 space group, and (*b*) the *Pc* setting along with the equivalent but non-standard *Pn* and *Pa* settings.

By default, the symmetry group visualizer will show the standard setting for a space group, but all the available settings for the selected group are available in a drop-down selection box at the bottom of the page. The display and list of symm­etry elements will update as needed when a different setting is selected. There is also the option in the drop-down menu to select and overlay representations of multiple settings. Two illustrative examples displaying multiple settings for the *R*3 and *Pc* space groups are shown in Fig. 3 ▸.

The symm­etry elements for all settings are listed in the panel on the right-hand side. This allows direct com­parison, showing, for example, that there is no centering vector in the rhombohedral setting of *R*3 and the *c*-, *n*-, and *a*-glide planes in the *Pc*, *Pn*, and *Pa* settings are all equivalent.

#### Plane and other groups

**Figure 4:**
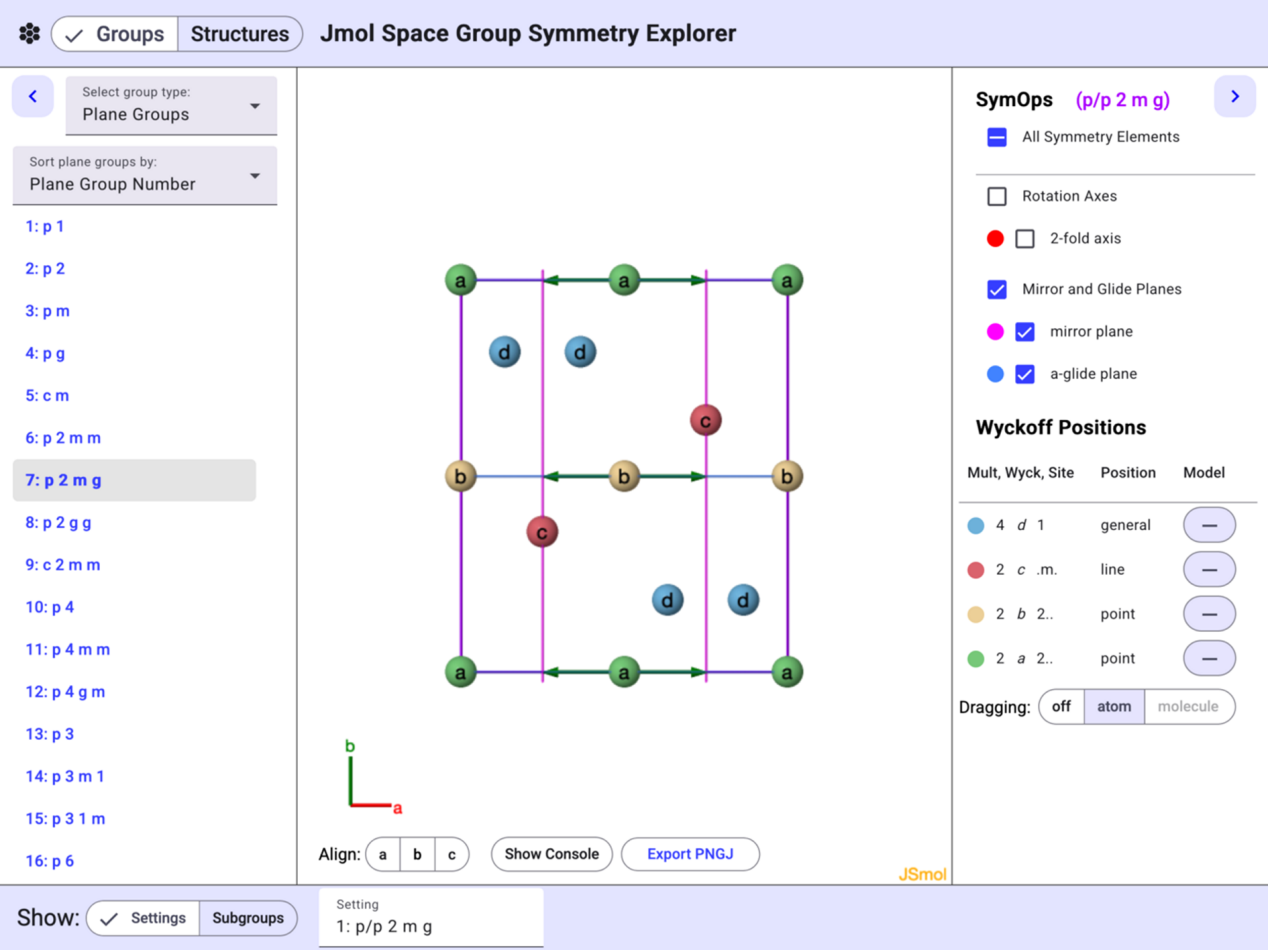
The *p*2*mg* plane group showing mirror planes, glide planes, and Wyckoff positions. Note: twofold axes are omitted so as to not obscure the Wyckoff labels.

All of the features described above are also available for any of the plane, frieze, rod, and layer groups. These simpler representations can be particularly useful in an educational context as stepping stones to the more com­plex space groups. An illustration of the *p*2*mg* plane group is shown in Fig. 4 ▸ with spheres representing the available Wyckoff positions.

**Figure 5:**
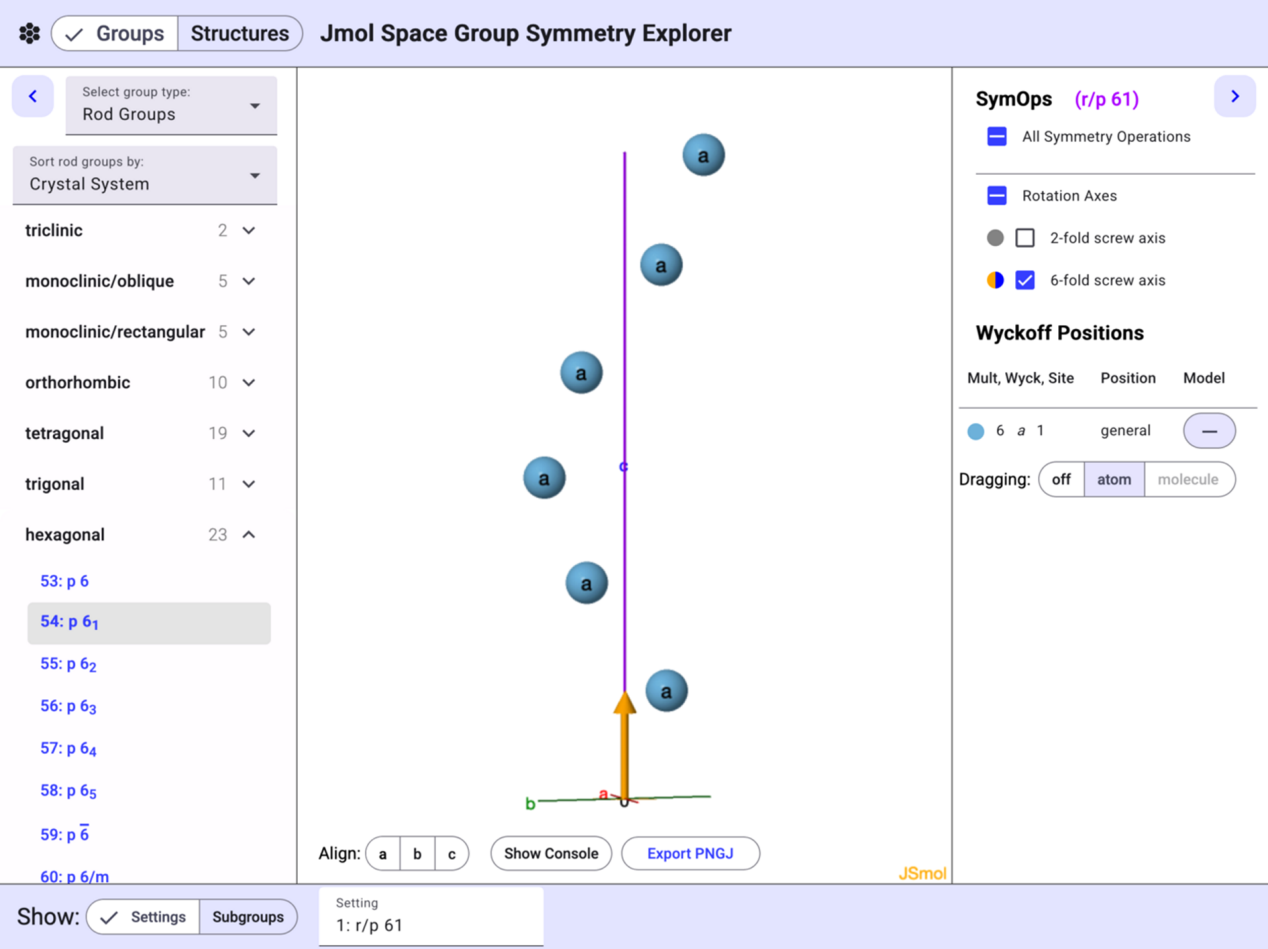
The *p*6_1_ rod group with a set of atoms representing general positions. Note that the rod group is periodic only in the *c* direction. The 3D coordinates of the atoms are constrained by the sixfold screw axis.

Another representation that is potentially valuable for educators is illustrated in Fig. 5 ▸, showing the rod group *p*6_1_ and a representation of the corresponding general position. The 6_1_ screw axis is clearly shown and the pseudo­atoms can be dragged, with all other symmetry-equivalent positions updating to reflect the underlying symm­etry. Other symm­etry relationships can be readily illustrated using similar plane, rod, and layer groups.

#### Maximal subgroups

**Figure 6:**
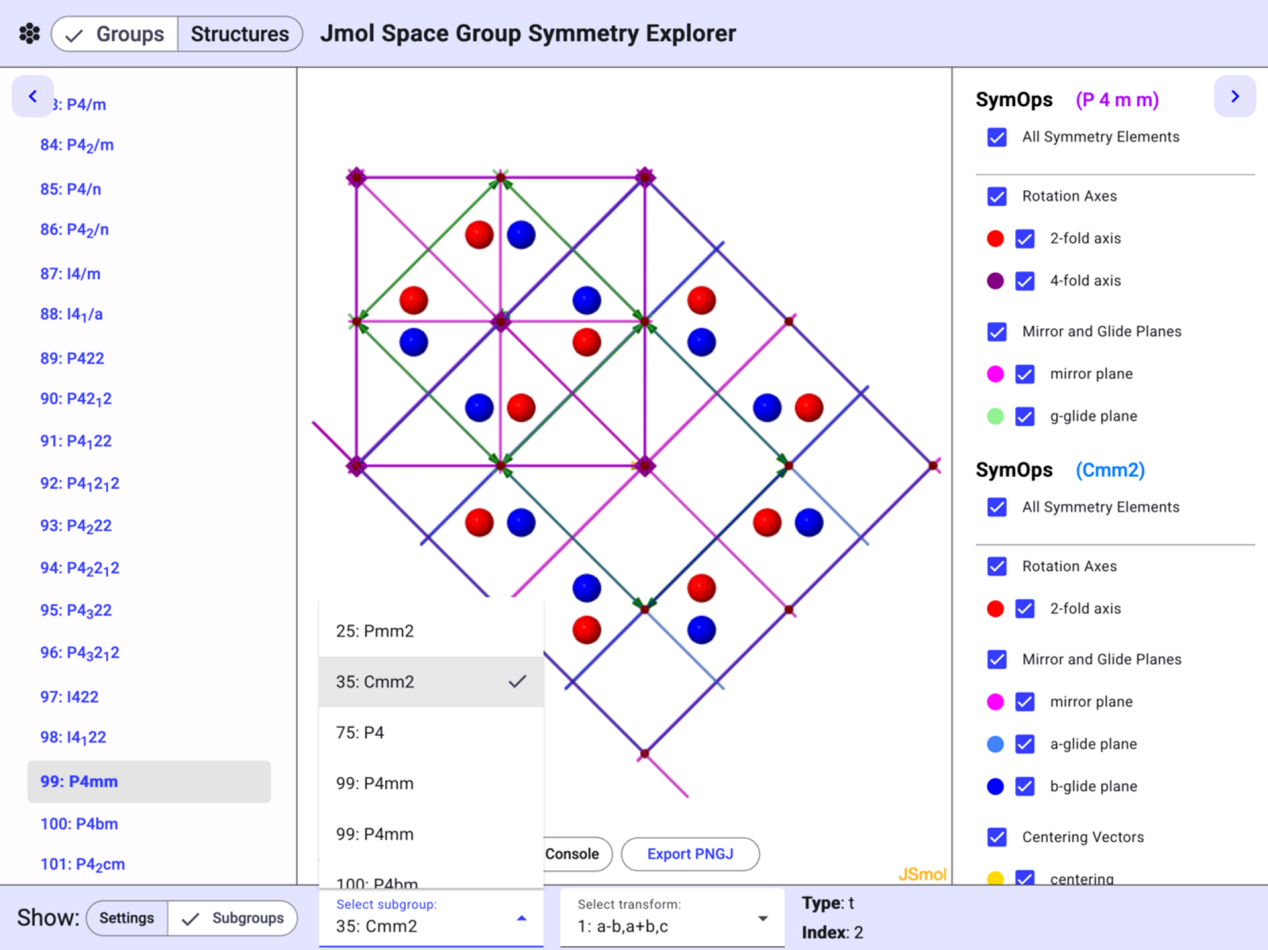
The *P*4*mm* to *Cmm*2 (99 > *a*–*b*,*a*+*b*,*c* > 35) group–subgroup transformation.

*Jmol* is also capable of generating lists of space group–subgroup relationships and displaying informative representations of the resulting symm­etry relationships. Switching the toggle bar at the bottom left of the website to subgroups will display a list of the maximal subgroups of the currently selected group. Selecting one of the subgroups will generate an overlay of the two groups, with pseudo­atoms at general positions representing the symm­etry of the original group and colored according to their symm­etry in the subgroup. An example group–subgroup relationship, 99 > *a*–*b*,*a*+*b*,*c* > 35 in CLEG notation (Hanson, 2025*a* ▸), is shown in Fig. 6 ▸.

### Structure symm­etry visualizer

**Figure 7:**
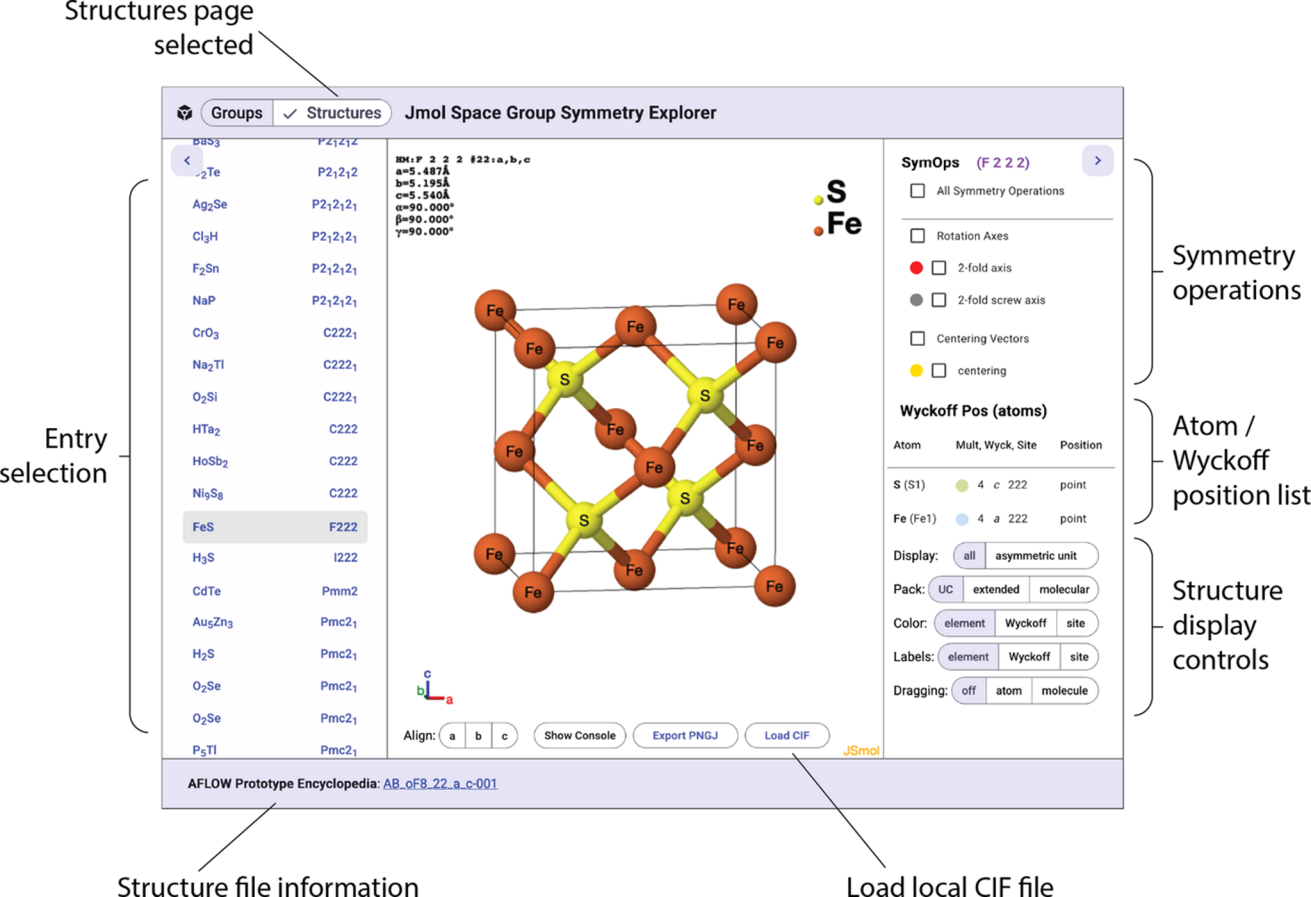
An annotated view of the *Jmol***Structure Symmetry Visualizer** displaying the structure of FeS (space group *F*222).

The layout of the structure symm­etry visualizer (see Fig. 7 ▸), selected using the toggle bar at the top left, is similar to the group visualizer. On the left is the list of structures, the *Jmol* display is in the center, and information and controls are on the right. Two sources of structural data are available. The first option is the AFLOW library of crystallographic prototypes (Mehl *et al.*, 2017 ▸). This set includes the option to filter the list by number of unique elements, making it possible to limit the list to elemental crystal structures or binary salts, for example. The second set com­prises a curated set of representative structures taken from the Cambridge Structural Database (CSD; Groom *et al.*, 2016 ▸). Entries were selected by searching for representative structures from each space group and prioritizing structures with a minimal number of atoms in the asymmetric unit.

Symmetry elements, off by default, can be selectively dis­played using the checkbox controls in the right panel. Additional information includes a list of atoms in the asymmetric unit, along with their respective Wyckoff positions. The packing controls fill the unit cell, the unit cell extended by closest neighbors, or with mol­ecules within the unit cell. Atoms can be colored and/or labeled by element, by Wyckoff position, or by site symm­etry. Depending on the type and symm­etry of the structure, mol­ecules or atoms of the structure can be dragged and the position of symm­etry-related mol­ecules or atoms will be updated in real time. Information about the structure and links to the respective database are shown in the bottom toolbar.

**Figure 8:**
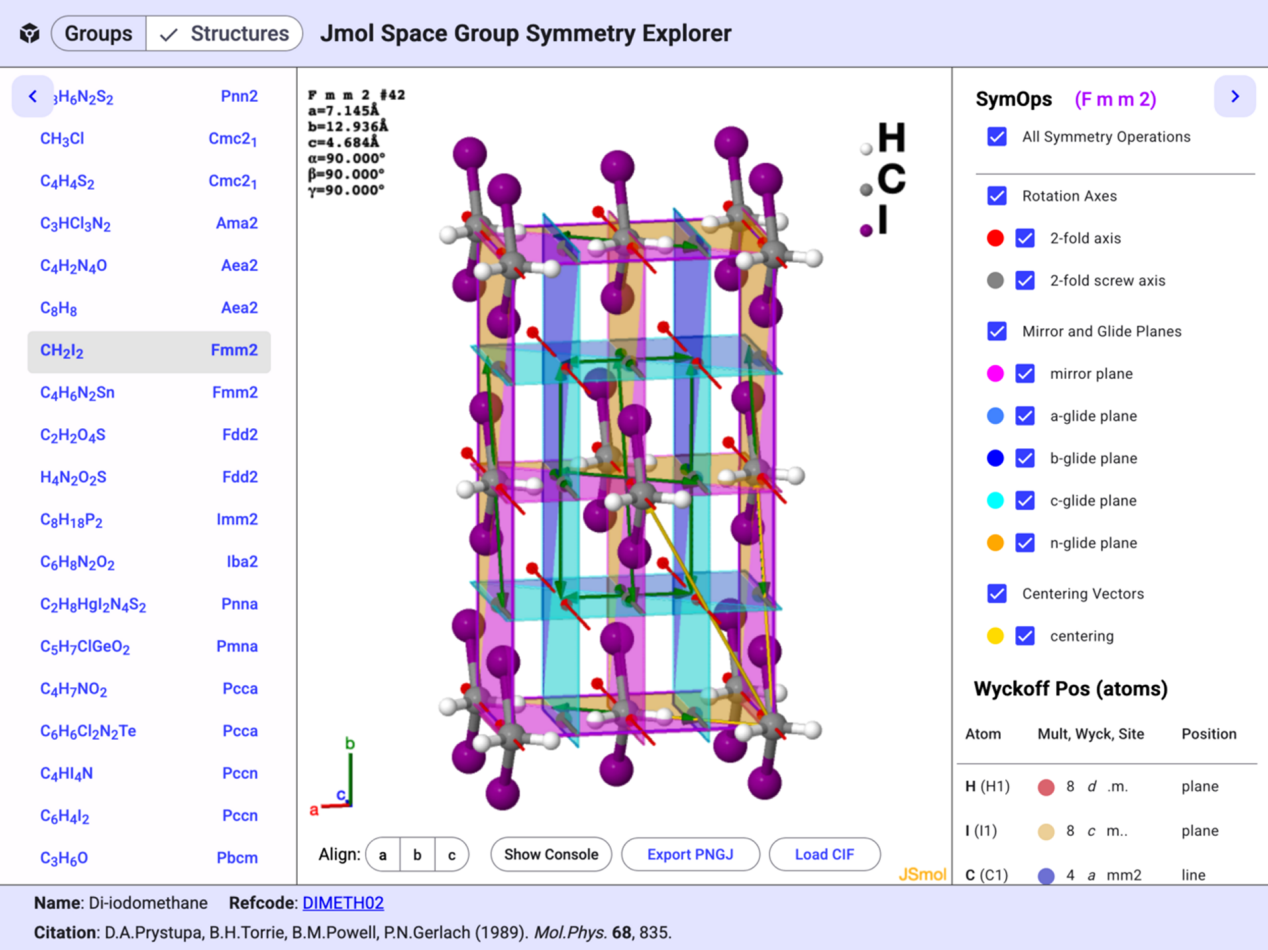
The unit cell of di­iodo­methane (space group *Fmm*2, CSD entry DIMETH02), illustrating the rotation axes, mirror and glide planes, and centering vectors.

An example mol­ecular structure, di­iodo­methane, entry DIMETH02 in the CSD (Prystupa *et al.*, 1989 ▸), is illustrated in Fig. 8 ▸. The space group is *Fmm*2, with the C, H, and I atoms each occupying a unique Wyckoff position. The mirror and glide planes are all clearly identifiable using the legend in the right panel. The C atoms are located on the twofold rotation axes at the inter­sections of the perpendicular mirror planes, corresponding to Wyckoff position *a*.

**Figure 9:**
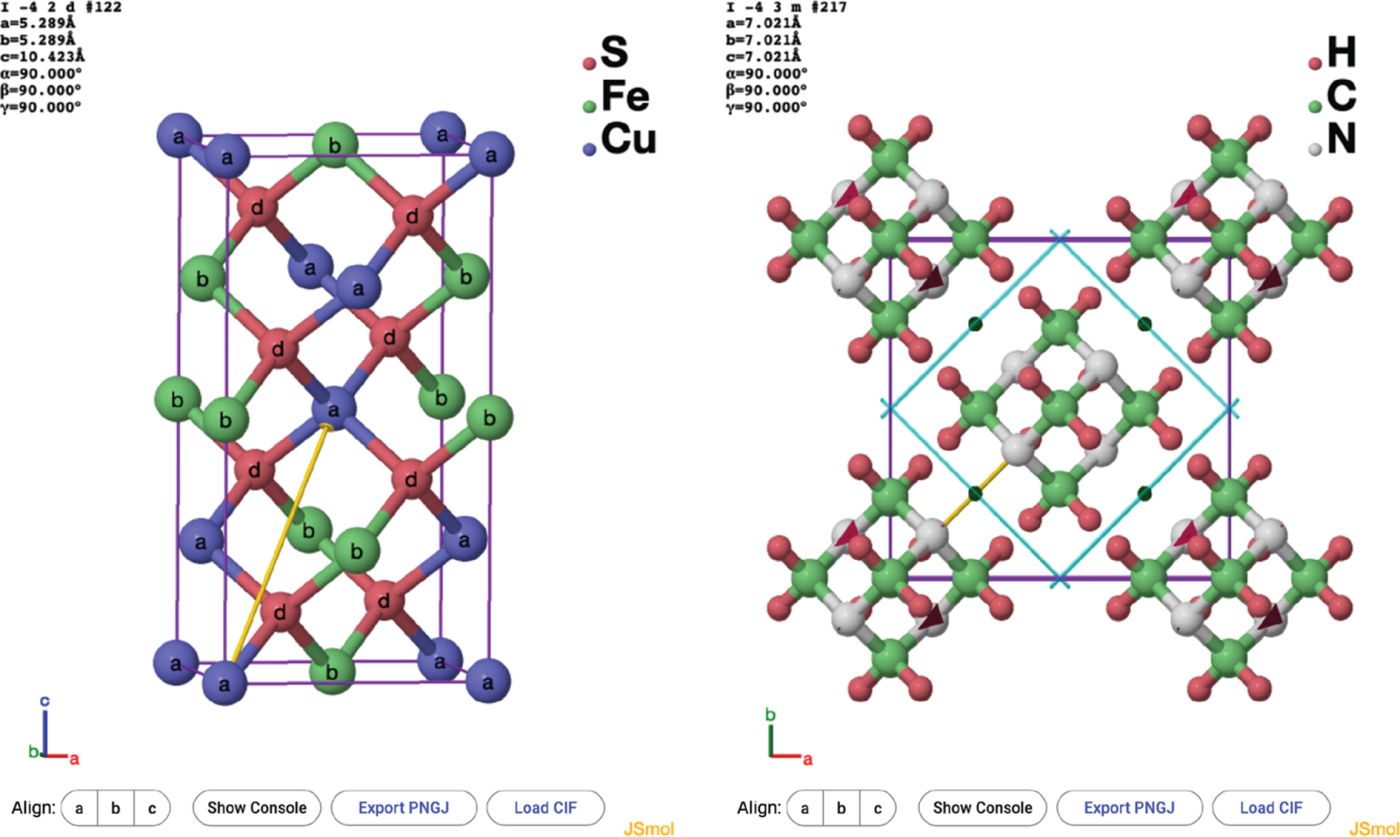
Two different structures, (*a*) FeCuS_2_ (space group *I*
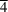
2*d*) and (*b*) hexa­methyl­ene­tetra­mine (space group *I*
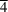
3*m*), each with the atoms colored and labeled by Wyckoff position.

The option to color atoms by Wyckoff position is highlighted in Fig. 9 ▸ with the structures of (*a*) FeCuS_2_ and (*b*) hexa­methyl­ene­tetra­mine.

#### Reading local CIF files

The existing website includes nearly 2000 example structures, but it can also load and display structures from local CIF files. The ‘Load CIF’ button at the bottom of the *Jmol* display provides a prompt from which the user can select a local file. The file will be loaded into the site, the structure dis­played, and the symm­etry elements and atom list with Wyckoff information will be shown in the right panel. Alternatively, users can just drag and drop a CIF file onto the *Jmol* display of the web page and the structure will load and display the relevant information and controls.

### Additional export and sharing features

#### PNG file export

*Jmol* has the capability to generate a special form of png (portable network graphic) file that is both a capture of the current display *and* the full *Jmol* state. When these files are opened or dropped onto a *Jmol* instance in a web page or opened with the stand-alone Java *Jmol* pro­gram everything in the current structure/representation will be regenerated.

#### URL sharing

The website is designed to provide identifying information for the current model or structure in the browser address bar so users can share a particular space group or structure by simply copying the current URL.

## Examples and tutorials

A set of examples and tutorials has been created and is available as part of the supporting information associated with this article. These materials include:

– Step-by-step inter­active tutorials covering inversion, reflection, rotation, rotoinversion, screw, and glide operations. Selected rod groups are used to focus on a single operation/element in each case. Inter­active dragging of pseudo­atoms helps reinforce the symm­etry relationships between sets of atoms.

– Annotated examples of plane groups and space groups, building on the representations introduced in the earlier tutorial. The addition of two- and three-dimensional motifs help emphasize the transformations resulting from glide and screw operations.

– Three examples illustrating Wyckoff positions and their site symmetries. The tutorials include examples of pseudo­atoms that are positionally constrained to a plane, line, or point, emphasizing the connection to the site description in the corresponding table.

– Two structural examples, one mol­ecular, one ionic, illustrating Wyckoff positions and the asymmetric unit.

– Three examples illustrating space group–subgroup relationships.

We hope that these examples serve to illustrate just a few of the ways that this website can be used to inform and inspire students to learn and practice crystallography.

## Conclusions

The *Jmol***Space Group Symmetry Visualizer** website com­bines the crystallographic capabilities of *Jmol* with a web inter­face to create a flexible resource for the visualization of symm­etry in crystallography. These materials provide a modular set of accessible and intuitive resources for students and faculty inter­ested in the teaching and learning of crystallography and crystallographic symm­etry.

## Supplementary Material

Jmol Space Group Symmetry Explorer: Tutorials and Examples. DOI: 10.1107/S2053229626004559/ky3232sup1.pdf
