# Supplementary material for: Visualizing and teaching crystallographic symm­etry using Jmol
Source: Acta Crystallogr C Struct Chem. 2026 May 18;82(Pt 6):258–66. doi: 10.1107/S2053229626004559 (PMC13237484; doi:10.1107/S2053229626004559)

Jmol Space Group Symmetry Explorer: Tutorials and Examples

I. Symmetry Operations (without translation) 2

A. Inversion 2

B. Reflection 2

C. Rotation 3

D. Rotoinversion 3

II. Symmetry Operations (with translation) 4

A. Screw 4

B. Glide 4

III. Plane Groups and Space Groups 5

IV. Wyckoff Positions 8

V. Symmetry Operations, Wyckoff Positions, and the Asymmetric Unit 9

A. Molecular structure 9

B. Ionic structure 10

VI. Maximal Subgroups 11

A. Pm > a,b,c > P1 *.......... 11*

B. P2/c > a,b,c > Pc *.......... 11*

C. ***C2/m > a,b,c > P2/m*** *.......... 12*

I. Symmetry Operations (without translation)

A full description of the symmetry of crystalline materials requires the use of **space groups**, a way to describe the full range of symmetry relationships in 3D space. To simplify the visual representation of these symmetry elements and operations, some examples will be displayed using **rod groups** and **plane groups**, 1D and 2D representations of periodic symmetry.

A. Inversion

**Operation:** inversion	**Symbol:** $\bar{1}$	**Element:** inversion center	**Representation:** yellow sphere

The inversion operation happens with respect to an *inversion center*. If we take the inversion center to be the origin, then the signs of each of the coordinates of any objects are reversed, effective moving that object an equal distance to the other side of the inversion center.

- Go to **Rod Group p-1** (2): spacegroups.symotter.org/group/r/2
- Select **Align** along **b**
- Add an atom in a *general position* by clicking the plus sign (+) in the line labeled **general**
- Click and drag on one of the blue atoms (labeled *c*). What is the relationship between the two atoms with respect to the inversion center?
- *Rotate* the model by clicking and dragging (away from the atoms) or *align* the model by clicking on any of the align options.
- Alternatively, turn on the **3D Motif**. Notice how the "handedness" of the tetrahedral motif is inverted.

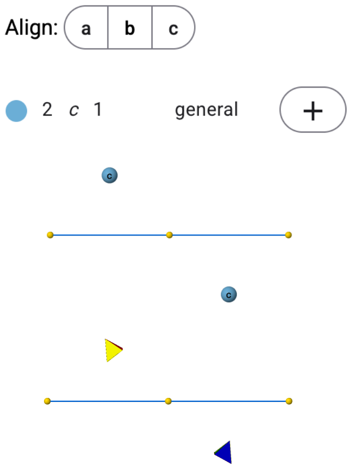

B. Reflection

**Operation:** reflection	**Symbol:** m	**Element:** mirror plane	**Representation:** pink plane

The reflection operation occurs with respect to a *mirror plane*. The mirror image of any object is generated an equal distance on the other side of the mirror plane.

- Go to **Rod Group pm** (4): spacegroups.symotter.org/group/r/4
- Click and drag to rotate the model and observe the representation of the reflection plane (pink plane).
- Add an atom in a *general position* by clicking the plus sign (+) in the line labeled **general**
- Click and drag on one of the blue atoms (labeled *b*). What is the relationship between the two atoms with respect to the mirror plane?
- Alternatively, turn on the **3D Motif**. Again, notice how the "handedness" f the tetrahedral motif changes upon reflection.

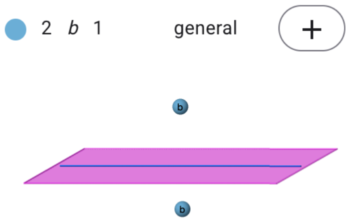

C. Rotation

**Operation: rotation**	**Symbol:** 2,3,4,6	**Element:** rotation axis	**Order**	**Representation**
○ **Rod Group p2** (3): spacegroups.symotter.org/group/r/3			2-fold (180°)	red arrow
○ **Rod Group p3** (42): spacegroups.symotter.org/group/r/42			3-fold (120°)	brick red triangle
○ **Rod Group p4** (23): spacegroups.symotter.org/group/r/23			4-fold (90°)	purple square
○ **Rod Group p6** (53): spacegroups.symotter.org/group/r/53			6-fold (60°)	purple hexagon

The rotation operation occurs around a *rotation axis*. In crystals (materials with translational symmetry) only 2-, 3-, 4-, and 6-fold rotations are allowed.

- Select one of the links in the table above
- Click and drag to rotate the model so that the axis is not pointing directly toward the viewer
- Add an atom in a *general position* by clicking the plus sign (+) in the line labeled **general**
- Click and drag on one of the blue atoms. What is the relationship between the set of (n) atoms with respect to the n-fold axis?

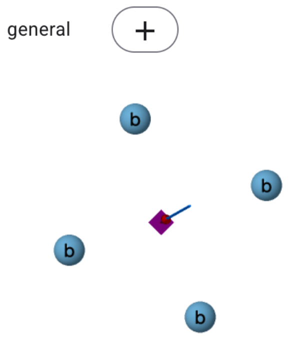

D. Rotoinversion

**Op: rotoinversion**	**Symbol:** $\bar{3}, \bar{4}, \bar{6}$	**Element:** rotation axis	**Order**	**Representation**
○ **Rod Group p-3** (45): spacegroups.symotter.org/group/r/45			3-fold (120°)	brick red triangle/arrow
○ **Rod Group p-4** (27): spacegroups.symotter.org/group/r/27			4-fold (90°)	purple square/arrow
○ **Rod Group p-6** (59): spacegroups.symotter.org/group/r/59			6-fold (60°)	purple hexagon/arrow

The roto-inversion operation is essentially the combination of an n-fold rotation (about a *rotation axis*) followed by *inversion* through an inversion point located on the rotation axis. Note that a $\bar{1}$ operation is rotation by 360 degrees followed by inversion, or just inversion. The $\bar{2}$ operation is equivalent to a mirror plane.

- Select one of the links in the table above
- Click and drag to rotate the model so that the axis is not pointing directly toward the viewer
- Add an atom in a *general position* by clicking the plus sign (+) in the line labeled **general**
- Click and drag on one of the blue atoms. What is the relationship between the set of atoms with respect to the *roto-inversion* axis?

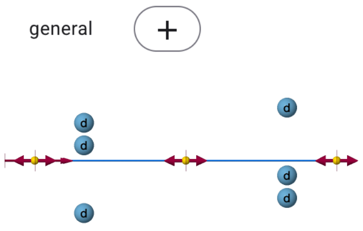

II. Symmetry Operations (with translation)

A. Screw

**Operation:** screw	**Symbol:** n_m	**Element:** screw axis	**Order**	**Representation**
○ **Rod Group p6(1)** (54): spacegroups.symotter.org/group/r/54			6-fold : trans 1/6	yellow 6-pinwheel
○ **Rod Group p6(2)** (55): spacegroups.symotter.org/group/r/55			6-fold : trans 2/6	yellow 3-pinwheel
○ **Rod Group p6(5)** (58): spacegroups.symotter.org/group/r/58			6-fold : trans 5/6	blue 6-pinwheel

The *screw* operation combines a rotation with a translation of some fractional distance along a unit cell vector. The operation is denoted n_m with a rotation of $2\pi/n$ and a translation of m/n (fractional translation). They can be visualized as they are named – very much like the threads on a screw or a spiral staircase. The 6_1 and 6_5 screw axes generate the same spirals, just in opposite direction.

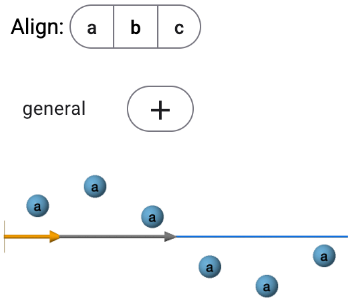

- Select one of the links in the table above
- Select **Align** along **b**
- Add an atom in a *general position* by clicking the plus sign (+) in the line labeled **general**
- Click and drag on one of the blue atoms. What is the relationship between the set of atoms with respect to the *roto-inversion* axis?
- Try some of the other rod groups listed above, or any of the set from p6_1 through p6_5 – how are they similar? How are they different?

B. Glide

**Operation:** glide	**Symbol:** *a*, *b*, *c*, *n*, *e*, *d*	**Element:** plane	**Representation:** translucent plane

The *glide reflection* operation combines reflection with translation parallel to the mirror plane. The planes denoted *a*, *b*, and *c* translate along the respective cell axes.

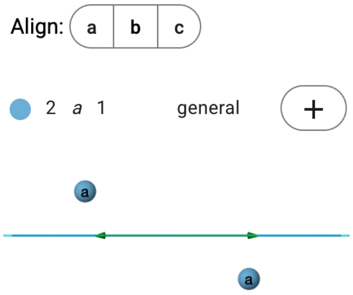

- Go to **Rod Group pc** (5): spacegroups.symotter.org/group/r/5
- Select **Align** along **b**
- Add an atom in a *general position* by clicking the plus sign (+) in the line labeled **general**
- Notice that this is a *c*-glide. The translation is along the *c* axis.
- Click and drag on one of the blue atoms. What is the relationship between the two atoms with respect to the glide plane?

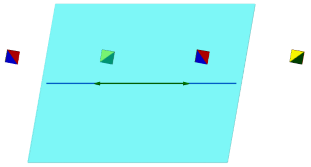

- Alternatively, turn on the **3D Motif** (viewed along ***a***). Notice how the "handedness" of the tetrahedral motif is inverted by the reflection operation. Rotate the model to see the effect of the glide operation.

III. Plane Groups and Space Groups

The full symmetry of a periodic structure (crystal) can be described using a collection of one or more symmetry operations known as the *space group*. You may have noticed that in some cases the presence of one symmetry operation requires the presence of additional operations. For example, if there is a $\bar{6}$ axis, we will also have a 3-fold axis (3) and a mirror plane (m) perpendicular to the rotation axis.

Plane groups, also known as wallpaper groups, are groups of symmetry operations that describe the symmetry of two-dimensional periodic arrangements. They are simpler to visualize because of their two-dimensional nature. Consider the examples below:

Plane Group: p2mm
Elements: 2-fold axis, perpendicular mirror planes
System: rectangular
Link: spacegroups.symotter.org/group/p/6
Notes: Notice the symmetry relationships between the atoms and the motif include both the mirror planes and the 2-fold axes. Drag one of the blue atoms and note how they maintain their relative positions. The triangles can be generated by selecting the **2D Motif** on/off option box.

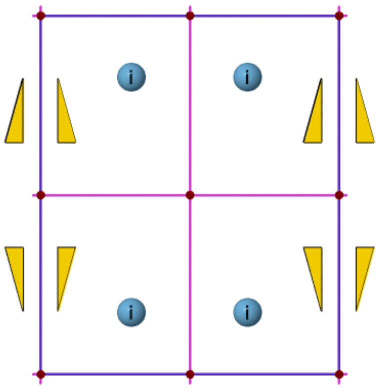

Plane Group: p2gg
Elements: 2-fold axis, perpendicular glide planes
System: rectangular
Link: spacegroups.symotter.org/group/p/8
Notes: How is this different from the p2mm plane group? Notice how the 2-fold axes are in the same positions, but the glide planes are at ¼ and ¾ along the a and b axes. The double-headed arrows represent the glide translation.

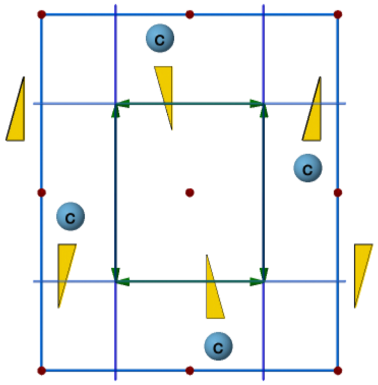

Plane Group: p6
Elements: 6-fold, 3-fold, 2-fold axes
System: hexagonal
Link: spacegroups.symotter.org/group/p/16
Notes: Note how each 6-fold axis will include a 3-fold and 2-fold axis at the same position. There are additional 3-fold and 2-fold axes generated within the unit cell by the combination of the 6-fold axis and translation along the *a* and *b* directions.

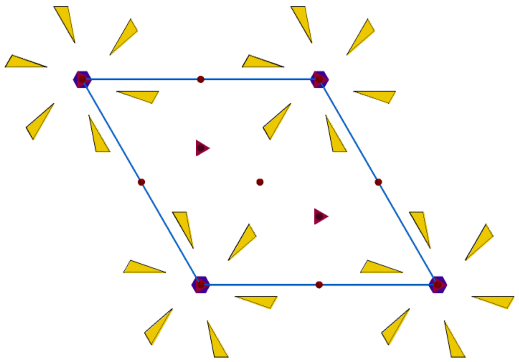

Space Group: $P2_1/c$ (number 14)
Elements: 2_1 screw axes, c-glide, inversion centers
System: monoclinic
Link: spacegroups.symotter.org/group/s/14
Notes: The images on the right show the $P2_1/c$ space group viewed down the *b* axis with and without the *c*-glide planes.

The 2_1 screw axes are aligned with the *b*-axis (towards the viewer).

Note that the screw axis does not invert the handedness of the tetrahedral motif, while both the glide plane and the inversion center do.

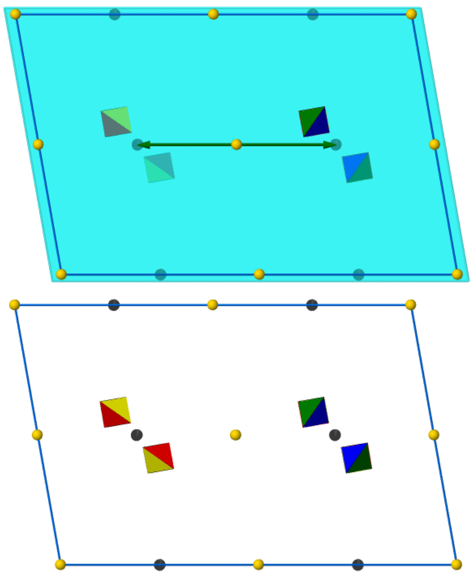

Space Group: $P2_12_12_1$ (number 19)
Elements: 2_1 screw axes
System: orthorhombic
Link: spacegroups.symotter.org/group/s/19
Notes: The image on the right show the $P2_12_12_1$ space group viewed down the *c* axis. The 2_1 axes run in all three directions. Can you see how each of the motifs is related to its counterpart *via* a 2_1 screw running along each of the axes?

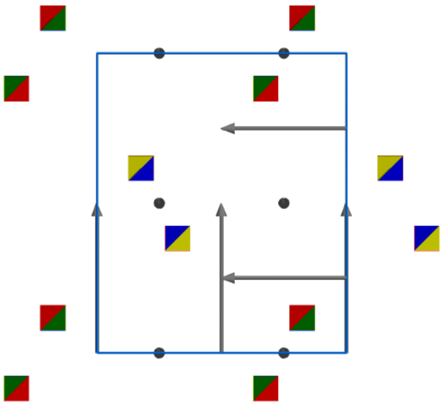

Space Group: Pbca (number 61)
Elements: *a*,*b*,*c*-glide, 2_1 screw, inversion centers
System: orthorhombic
Link: spacegroups.symotter.org/group/s/61
Notes: The images on the right show the Pbca space group viewed down the *c* axis with the a-glide plane hidden for clarity.

The 2_1 axes run in all three directions as do the glide planes. The 2_1 axes are all contained within one of the glide planes.

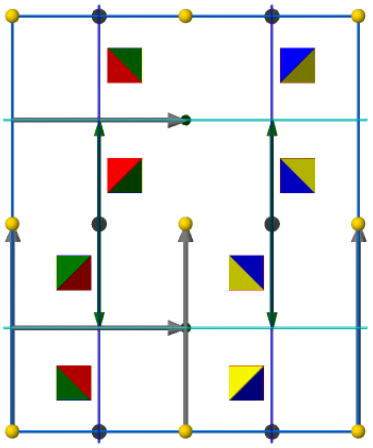

Space Group: P-31c (number 163)
Elements: *c*, *g*, *n*-glide; 2, 3, 2_1, $\overline{3}$ axes; inversion
System: trigonal
Link: spacegroups.symotter.org/group/s/163
Notes: The top image on the right shows the P-31c space group viewed down the *a* axis (*c*-glide, *g*-glide planes and 2_1 axes hidden). Note how the *n*-glide translation does not align with any of the primary axes.

The bottom image on the right shows the P-31c space group viewed down the *c* axis. The $\overline{3}$ axes sit on the corners with additional 3-fold axes in the center of the unit cell.

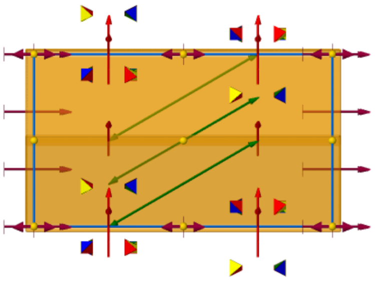

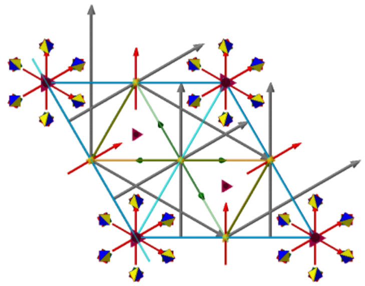

IV. Wyckoff Positions

Plane Group p2mg (number 7)

Load the **p2mg** plane group: spacegroups.symotter.org/group/p/7.

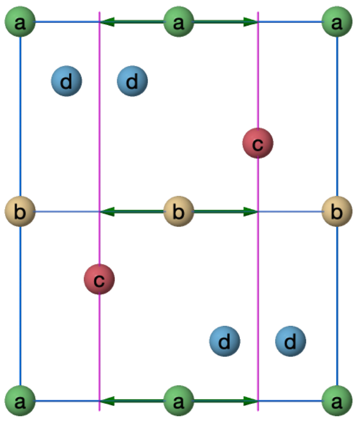

- Click on all of the **+** signs in the **Wyckoff Positions** table to add pseudo-atoms for all of the positions in the p2mg plane group.
- Click and drag the **d** atoms (in the *general position*). Are there any restrictions on where these atoms can be placed?
- Now try dragging an atom labeled **c**. How are their positions restricted? Consider the symmetry operations in the group – how are the two **c** atoms related by symmetry?
- Look at the label in the Wyckoff table: "**.m.**" This notation indicates that this position is constrained to the mirror plane in the *b* direction (the *b* direction is the secondary axis)
- Click and drag one of the **a** or **b**-labeled atoms. How are their positions constrained? Their label (**2..**) indicates that they sit on one of the 2-fold axes along the primary (*c*) axis. The position column is labeled **point**, meaning that these Wyckoff Positions are constrained to specific points within the unit cell.

Space Group P2$_1$/c (number 14)

Load the **P2$_1$/c** space group: spacegroups.symotter.org/group/s/14.

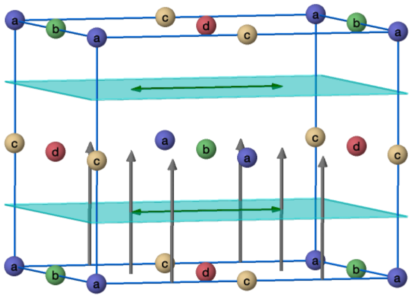

- Click on the **+** signs in the **Wyckoff Positions** table to add pseudo-atoms for **a**, **b**, **c**, and **d** positions.
- Click and drag the **d** atoms (in the *general position*). Are there any restrictions on where these atoms can be placed?
- Notice that if you try and drag any of these atoms that they are fixed. Each of these Wyckoff Positions is located on one of the inversion centers (-1) within the unit cell, as indicated in the Wyckoff Positions table.

Space Group P4$_2$2$_1$2 (number 94)

Load the P4$_2$2$_1$2 space group: spacegroups.symotter.org/group/s/94.

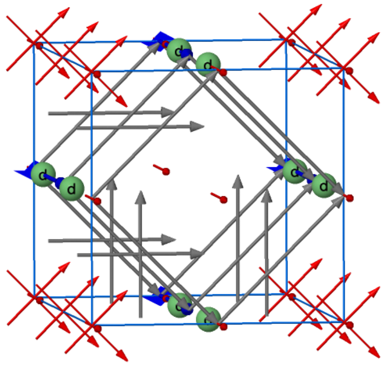

- Click on the **+** sign for the **d** position in the **Wyckoff Positions** table.
- Click and drag one of the **d** atoms. How is the movement of this position restricted?
- The **d** position is labeled **2..** indicating, like the **p2mg** example above, that the position is restricted to the 2-fold axis along the primary axis, which is the *c*-axis in a tetragonal system.
- Now add an atom in the **f** position. It is also constrained to lie along a 2-fold axis, but along the [110] and [1-10] directions, the secondary axis 2 for this space group.

V. Symmetry Operations, Wyckoff Positions, and the Asymmetric Unit

A. Molecular structure

- Load the structure of phosphinic acid (H_3PO_2), CSD refcode GALCAB04: spacegroups.symotter.org/structure/csd/GALCAB04

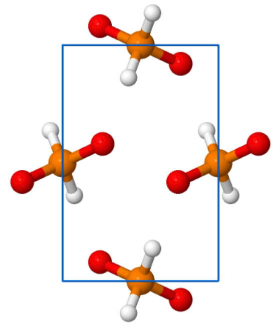

- Set **Dragging** to **molecule**

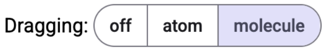

- Click and drag any molecule and observe how the molecule moves and how the other molecules respond. Is the movement of the molecule restricted in any way?
- Turn on the symmetry element representations by selecting the **All Symmetry Elements** checkbox. Does the structure maintain these symmetry relationships as you drag a molecule around?

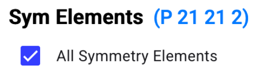

- Look at the **Wyckoff Positions** table. Does the site symmetry (**Position** column) help explain the restrictions on the movement of the molecule?

Atom	Mult, Wyck, Site	Position
**O** (O1)	4 *c* 1	general
**H** (H1)	4 *c* 1	general
**P** (P1)	2 *b* ..2	line

- Reload the page to reset the display.
- Change the **Display** to display the **asymmetric unit**. The *asymmetric unit* is the smallest unique part of a crystal structure that can be used generate the entire structure.

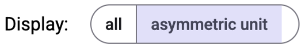

- The display now highlights the *three atoms* that define the entire structure (one oxygen, one hydrogen, and one phosphorous). Hopefully it is clear how the 2-fold and 2_1 screw axes define the other copies of the molecule. What symmetry relationship defines the “other half” of the molecule?

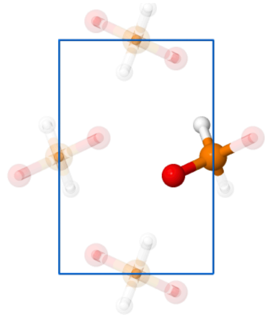

B. Ionic structure

- Load the structure of scheelite (calcium tungstate, $CaWO_4$), ICSD 60547 spacegroups.symotter.org/structure/aflow/AB4C_tI24_88_a_f_b-003

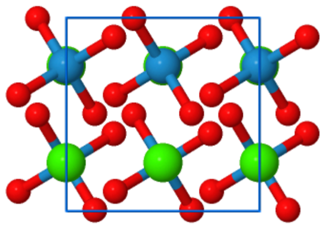

- Change the packing to **extended**

Pack: UC | extended | molecular

- Set **Dragging** to **atom**

Dragging: off | atom | molecule

- Click and drag any atom and observe how the structure changes. Are you able to drag any of the atoms, or only selected atoms?

- Look at the **Wyckoff Positions** table. Does the site symmetry (**Position** column) help explain the restrictions on the movement of the atoms?

Atom	Mult, Wyck, Site	Position
**O** (O1)	16 *f* 1	general
**Ca** (Ca1)	4 *b* -4..	point
**W** (W1)	4 *a* -4..	point

- Turn on the symmetry element representations for the 4-bar axes by selecting the **4-bar** axis checkbox.

Rotation Axes
- [ ] 2-fold axis
- [ ] 2-fold screw axis
- [ ] 4-fold screw axis
- [x] 4-bar axis

- Can you see how both the calcium and tungsten atoms (site **-4..** in the Wyckoff Table) are sitting on the 4-bar axes?

- Change the atom **Color** and atom **Labels** to **Wyckoff**. The atom colors and labels should now reflect the key given in the Wyckoff Table.

Color: element | Wyckoff | site
Labels: element | Wyckoff | site

- Change the **Display** to display the **asymmetric unit**.

Display: all | asymmetric unit

- The display now highlights the *three atoms* that define the entire structure (one oxygen, one calcium, and one tungsten). Try and determine what symmetry operations relate each of these three atoms to their counterparts in the unit cell. For example, the calcium atom (labeled **b**) is related to other calcium atoms by an inversion, the *a*-glide, and 4-fold screw.

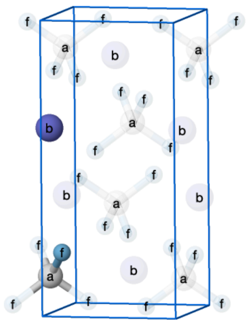

VI. Maximal Subgroups

Subgroups of space groups are often useful when studying solid-solid phase transitions, twinning, and structure prediction.

A. Pm > a,b,c > P1

- Load the Pm space group: spacegroups.symotter.org/group/s/6
- Add a *general position* pseudo-atom by clicking on the **+** next to the general label in the Wyckoff Position table. The resulting view should look like the image on the right (viewed down the *c* axis).

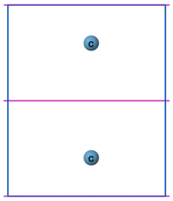

- Change the **Show** menu (bottom left) to show **Subgroups**

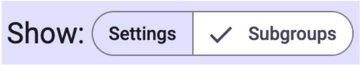

- From the **Select subgroup:** menu select the **1: P1** space group

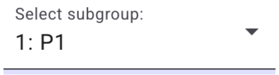

- The resulting diagram (shown on the right) illustrates that when we remove the mirror plane from the **Pm** space group to get the **P1** space group, we *split* the two equivalent positions from Pm into two distinct positions, denoted by two different colors. The transform in this case (a,b,c) retains the unit cell in its original position.

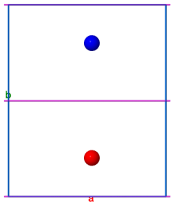

- Note: this is an example of a *t-subgroup*, defined as a subgroup where the *crystal class* changes (m -> 1)

A similar transformation (also a t-subgroup, 2/m -> m) is illustrated below (viewed along the *a*-axis) for removal of the 2-fold axis from the P2/c space group. Note how the *c*-glide is retained, but the 2-fold axis is not. The diagram on the right was generated as follows:

- Load the P2/c space group: spacegroups.symotter.org/group/s/13
- Change the **Show** menu (bottom left) to show **Subgroups**
- From the **Select subgroup:** menu select the **7: Pc** space group
- Select **Align** along ***a***

B. P2/c > a,b,c > Pc

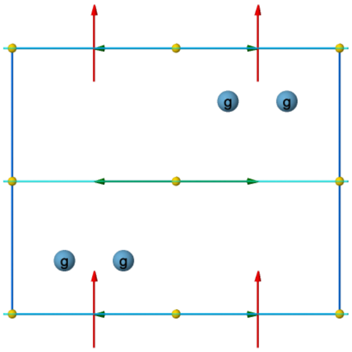

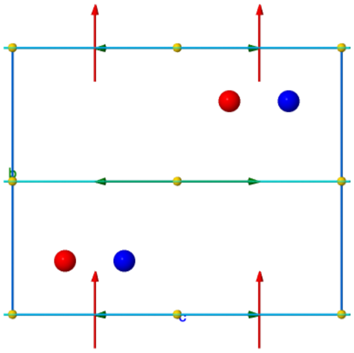

https://spacegroups.symotter.org/group/s/13?subgroup_idx=3&transform=1

C. C2/m > a,b,c > P2/m

- Load the C2/m space group: spacegroups.symotter.org/group/s/12
- Change the **Show** menu (bottom left) to show **Subgroups**

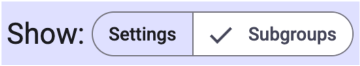

- From the **Select subgroup:** menu select the **10: P2/m** space group. Note: this represents a k-type transformation.

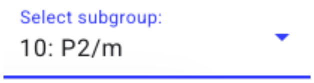

- Select **Align** along ***a***

- Look at the lists of symmetry elements for each of the two space groups. What elements are in the C2/m space group but not in P2/m?

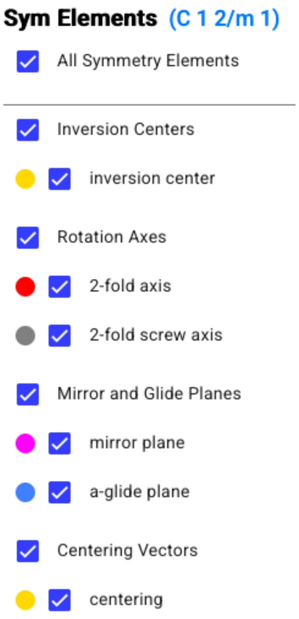

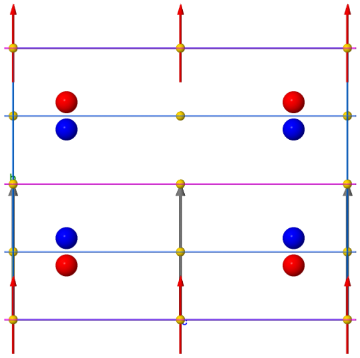

- Can you see how *all* the sites are related by symmetry in the top diagram, but only the matching colored atoms are related in the bottom figure?
- Rotate both versions to observe all symmetry relationships.

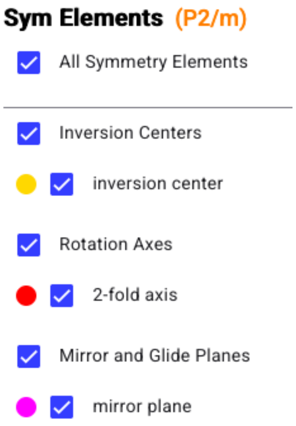

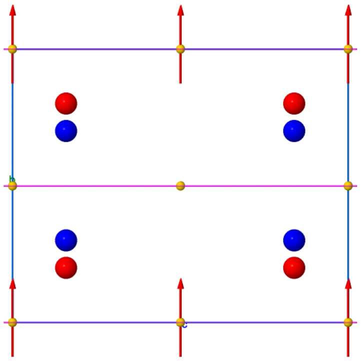

Supplement: Supplementary file 1 [file c-82-00258-sup1.pdf]
